# Oral Lichen Planus and Its Therapeutic Approaches: A Case Report

**DOI:** 10.7759/cureus.63192

**Published:** 2024-06-26

**Authors:** Mahek R Batra, Swapnil Mohod, Prem Sawarbandhe

**Affiliations:** 1 Department of Dentistry, Sharad Pawar Dental College And Hospital, Datta Meghe Institute of Higher Education and Research, Wardha, IND; 2 Department of Oral Medicine and Radiology, Sharad Pawar Dental College And Hospital, Datta Meghe Institute of Higher Education and Research, Wardha, IND

**Keywords:** oral lichen planus, malignant transformation, erythematous lesion, chronic inflammatory skin disorder, management approaches

## Abstract

Lichen planus, a chronic inflammatory skin disorder, presents with pruritic, polygonal, and flat-topped papules and plaques. It encompasses not only the skin but also mucous membranes, nails, and hair follicles. Diagnosis relies on all the clinical and biopsy reports. The etiology of oral lichen planus (OLP) is multifactorial, with genetic, immunological, and environmental factors playing significant roles. Frequently utilized therapies encompass topical corticosteroids, calcineurin inhibitors, and systemic immunomodulatory medications. Management should be tailored to disease severity and the specific site of involvement. Lichen planus can present in papular, hypertrophic, atrophic, erosive, or erythematous forms. In this report, we present a case of a 28-year-old male patient who presented with bilateral white striations on the buccal mucosa and an erythematous lesion on the right buccal mucosa causing significant discomfort. The patient was treated with corticosteroids, resulting in marked symptomatic relief and partial lesion regression over a follow-up period of six months. This case underscores the importance of early diagnosis and tailored therapeutic strategies in managing OLP to improve patient outcomes.

## Introduction

Lichen planus is a comparatively common mucocutaneous disorder that is mediated immunologically. It is a chronic disease and has an unpredictable course that alternates between remission and exacerbations. Clinically, six types of oral lichen planus (OLP) can be identified. Additionally, the condition can be autoimmune in its etiology [1].

Skin lesions can be disfiguring and in severe cases, there is involvement of oral or genital mucosa. Lesions from OLP have the potential to progress to squamous cell carcinoma. Moreover, occurrence in the scalp and the nails might also take place. Although most occurrences of lichen planus are idiopathic, some of them can be associated with habits (such as betel nut, smoking, and alcohol consumption); multiple disease processes and agents, such as viral and bacterial infections; autoimmune diseases; vaccinations; consumption of specific medications (for example, nonsteroidal anti-inflammatory drugs); or hepatitis C virus [2]. OLP histology is characterized by both sub-epithelial lymphohistiocytic infiltration and an abundance of enhanced intra-epithelial lymphocytes. In addition, there is degeneration of the basal keratinocytes leading to colloid or Civatte, hyaline, and cytoid bodies. The evidence based on ultrastructural observations of the colloid bodies implies that their nature is indeed apoptotic keratinocytes, further evidenced by more recent studies that used the end-labeling method to show DNA fragmentation within these cells [3-5].

OLP presents as white striations, erythema, erosions, papules, plaques, or blisters affecting predominantly the buccal mucosa, tongue, and gingivae. Patients may first complain of roughening of the oral mucosa, burning sensations, and discomfort in the oral mucosa when exposed to hot and spicy meals [6]. Squamous cell carcinoma may develop in less than 5% of OLP lesions in non-tobacco consumers. Therefore, OLP patients have an enhanced risk of developing oral cancer; it is uncertain whether it is a pre-malignant disorder [7,8].

## Case presentation

 A 28-year-old male presented to the Oral Medicine Department in Sawangi, Wardha, India, with a chief complaint of peeling mucosa from the right cheek mucosa for 15 days. The patient did not mention experiencing any previous injury or trauma to the affected region. There was no associated swelling, fever, or discharge. However, he also complained of pain and burning sensation aggravated while chewing and applying pressure on the affected area without any relieving factors as reported by the patient. The patient had no prior medical conditions and was not taking any medication. The patient did not have any prior dental treatment. The patient gave a habit history of chewing tobacco, lime, and betel nut.

**Figure 1:**
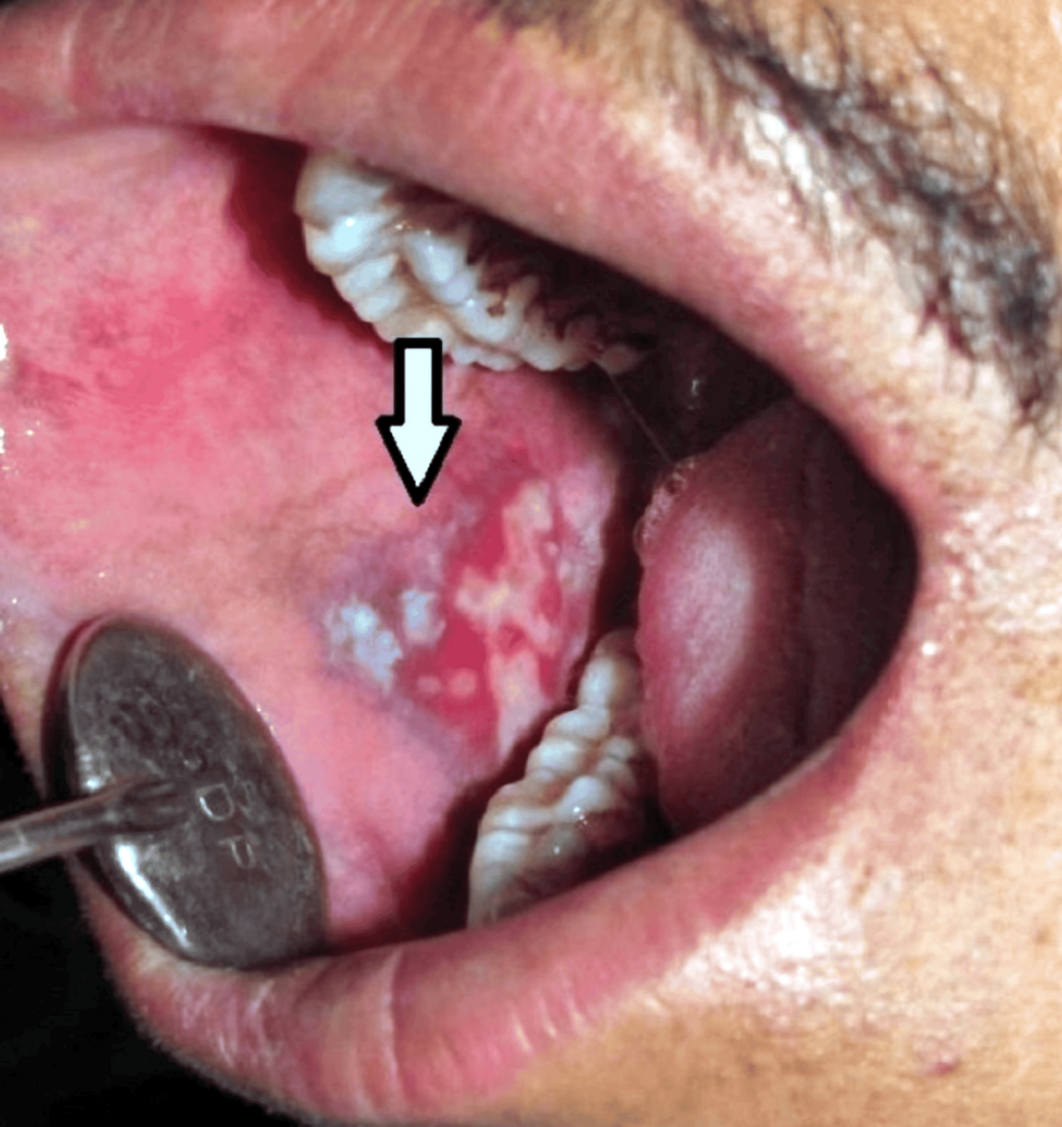
Intraoral examination of the patient showing mixed red and white lesion in the right buccal mucosa suggestive of erosive lichen planus (Informed consent from the patient was obtained before taking the photograph of the lesion) Image credit: Swapnil Mohod

**Figure 2:**
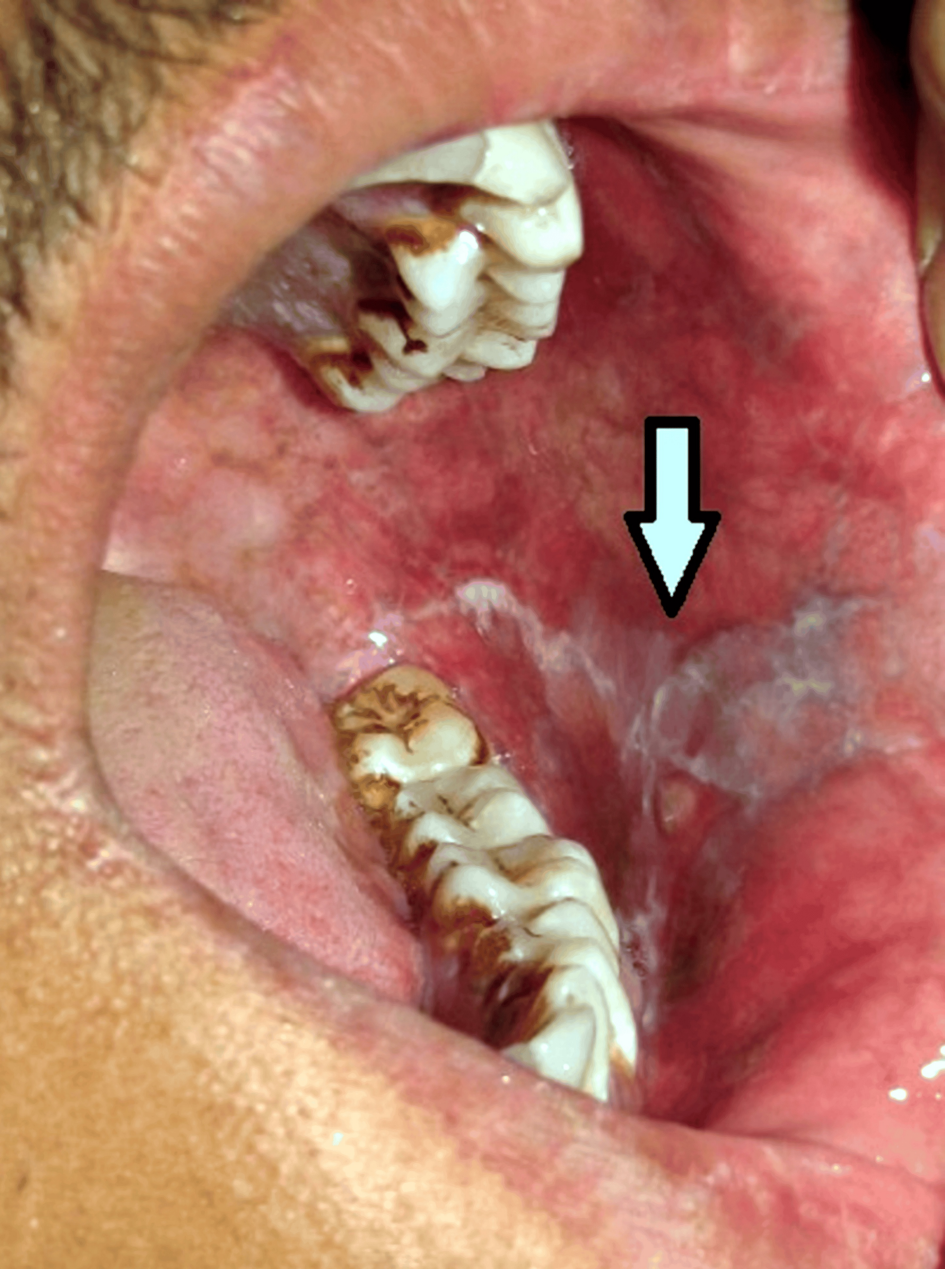
Intraoral examination of the patient showing reticular white striae in the left buccal mucosa suggestive of lichen planus Image credit: Swapnil Mohod

On intraoral examination, a whitish non-scrappable patch was seen on the right buccal mucosa with a reticular pattern and erythema seen extending from the retromolar region linearly to the left corner of the mouth. The red erythematous lesion was seen on the right buccal mucosa of size approximately 3 cm x 2.5 cm in 46, 47, and 48 regions with a white non-scrappable lesion on the right and left buccal mucosa as shown in Figures 1, 2.

The patient had overall poor oral hygiene, with bleeding upon probing and generalized gingival inflammation. Extraoral examination revealed a single lymph node in the left submandibular region palpable of size 0.5 cm x 0.5 cm, which was firm in consistency, mobile, and non-tender.

Treatment 

This case was treated with local application of triamcinolone acetonide 0.1% cream, and the patient was advised to apply it in a thin layer ensuring even coverage and gentle rubbing to aid absorption four times daily for seven days and also advised not to take any food or liquid for half an hour after application of the gel. The patient was prescribed systemic steroids (prednisolone) for only five days at a dose of 20 mg once a day after a meal.

The patient was recalled for review after five days and clinically the lesion size was reduced to 50% and the symptoms of the patient were reduced and was advised to avoid potential irritants such as spicy foods, alcohol-containing mouthwashes, and smoking, which could exacerbate symptoms, encourage a soft, bland diet to minimize irritation to the oral mucosa and promote healing.

The patient was asked to continue the topical application of triamcinolone acetonide 0.1% cream twice daily and the steroid dose was reduced to 5 mg once a day for the next 10 days. The patient emphasized good oral hygiene practices, including gentle brushing and flossing, to prevent secondary infections and maintain oral health. 

**Figure 3:**
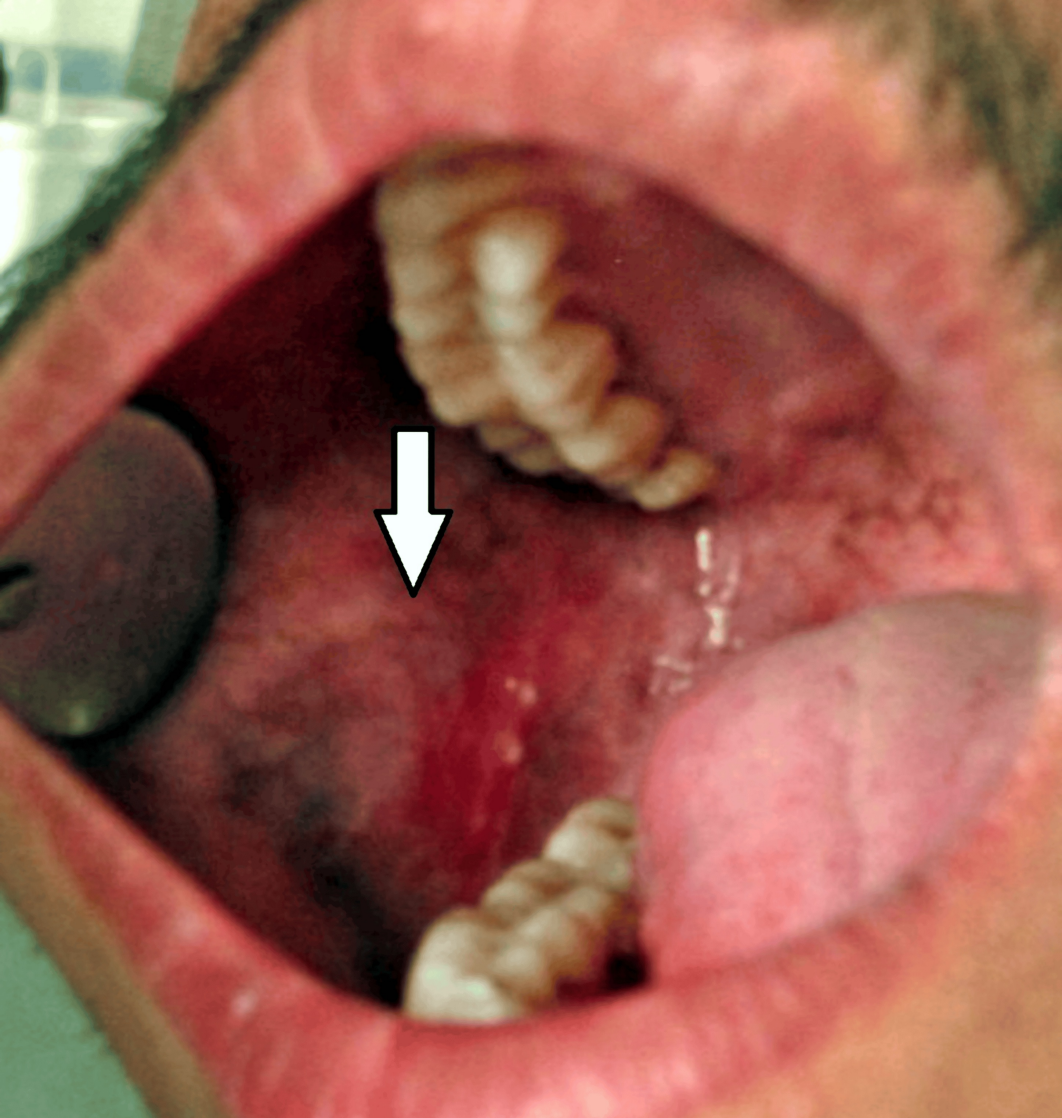
Post-treatment after 15 days, the patient experienced a complete cure, showing no remaining signs or symptoms Image credit: Swapnil Mohod

The patient recalled after 10 days for review, Figure 3 shows the post-treatment condition of the lesion, shows a complete reduction in size, and the patient had no discomfort and pain after 15 days. Later, the patient was recalled every month for six months.

## Discussion

Lichen planus is a chronic inflammatory skin disorder that presents with pruritic, polygonal, flat-topped papules, and plaques. It encompasses not only the skin but also mucous membranes, nails, and hair follicles. Diagnosis relies on all the clinical and histological findings [1]. Although most occurrences of lichen planus are of unknown cause, some of them can be associated with the consumption of specific medications or hepatitis C virus [2]. OLP demonstrated a greater incidence in women, typically appearing at a younger age in men, lacking familial predisposition, and it is frequently presented on the buccal mucosa and predominantly exhibited symptoms in its erosive form. The most prevalent form is the reticular type, distinguished by slender white lines (known as Wickham's striae) arising from the papules. Patients with reticular lesions are often not symptomatic, but erosive (ulcerative) or atrophic (erythematous) OLP is often associated with pain and burning sensation [9].

The assessment of oral lesions clinically relies on the six described clinical forms by Andreason [10]. The forms are papular, reticular, atrophic, plaque, bullous, and erosive. Mucosal lesions, when multiple, tend to show a balanced distribution, frequently found on the cheeks close to the molars and on the tongue lining. They are less commonly observed on the lips and gums, where the atrophic and erosive varieties might manifest as desquamative gingivitis. Even less frequently, they may appear on the palate and the floor of the mouth [11]. The most prevalent form is the reticular type, distinguished by slender white lines (known as Wickham's striae) emanating from the papules. Patients with reticular lesions are often asymptomatic, but erosive (ulcerative) or atrophic (erythematous) OLP is often associated with pain and burning sensation [12].

Performing a biopsy for every OLP lesion remains a subject of debate. In the case of papular, reticular, or plaque-like OLP lesions, if the clinical presentation is typical including bilateral and symmetrical lesions, the decision to confirm the clinical diagnosis through biopsy may or may not be taken. A biopsy is recommended for ulcerative, erosive, or bullous OLP lesions, particularly when there is a possibility of dysplastic alteration or the onset of malignancy is being considered. It is crucial to visually inspect and palpate OLP lesions during examination. If tissue induration is felt at the outer borders of an OLP lesion, a biopsy should be conducted as a precautionary measure to rule out any potential malignancy [12].

The treatment approach for OLP is subject to variation depending on the disease's particular forms and symptoms. In cases where patients display popular plaque-like OLP or reticular lesions without experiencing oral symptoms such as a burning sensation or pain when consuming acidic, spicy, hot, or salty foods, immediate treatment may not be necessary. All patients with OLP should have a complete awareness of the etiology and clinical aspects of the illness. Individuals experiencing bullous, erosive, ulcerative OLP commonly endure oral symptoms like burning sensations, pain, and inflammation. It is crucial to quickly administer effective drug treatments to these patients to ease their symptoms, so corticosteroids are the preferred choice for treatment. If the lesion is minor and the oral symptoms are modest, applying a thin film of corticosteroid ointment two or three times every day at most concerning regions is typically sufficient to facilitate the healing within two to three weeks. For bigger OLP lesions and moderate to severe oral discomfort, administering a corticosteroid powder spray on the afflicted mucosal regions two to three times a day is also useful in causing the lesions to heal. Treatment options for large and severe erosive oral lichen planus (EOLP) lesions include submucosal and intralesional injections of Kenacort-A and oral prednisolone administration (once daily for two weeks); the oral prednisolone administration is decreased to 5 mg per day and discontinued in the third week). Corticosteroid ointment can be applied topically to treat any mild lesions that may still be present [12].

For non-healing lesions, surgical excision is advised as it may offer a cure for the disease; however, it is not recommended for atrophic or erosive forms. Cryosurgery has also been employed in EOLP, although recurrences are frequent [10]. Patients should abstain from excessive work, severe exhaustion, sleeplessness, and hot, salty, acidic, or spicy foods. Even with effective corticosteroid treatment resolving symptoms, EOLP lesions tend to recur if patients' health declines. Factors like betel nut consumption, cigarette smoking, and alcohol consumption pose threats. Patients with EOLP should undergo reevaluations every three to six months, regardless of symptom presentation.

## Conclusions

Maintaining good oral hygiene is essential for the healing of OLP lesions, especially in the areas where lesions are present. Patients should refrain from extreme fatigue, hot, salty, acidic, or spicy foods as well as from working too much or sleeping too little. A mycostatin or other antifungal medication should be administered to patients for a minimum of two weeks to manage the candida infection, as prolonged maintenance use of corticosteroids has the potential to cause oral candidiasis. Patients with papular, reticular, or plaque-like OLP do not require treatment if they do not exhibit any oral symptoms. If patients' health deteriorates, EOLP lesions typically recur even after effective corticosteroid treatment. This recurrence can be triggered by factors such as betel nut consumption, cigarette smoking, and alcohol use. Patients taking EOLP should have reevaluations every three to six months, even if they show no symptoms.
